# *Monodopsis* and *Vischeria* Genomes Shed New Light on the Biology of Eustigmatophyte Algae

**DOI:** 10.1093/gbe/evab233

**Published:** 2021-10-19

**Authors:** Hsiao-Pei Yang, Marius Wenzel, Duncan A Hauser, Jessica M Nelson, Xia Xu, Marek Eliáš, Fay-Wei Li

**Affiliations:** 1 Boyce Thompson Institute, Ithaca, New York, USA; 2 School of Biological Sciences, University of Aberdeen, Aberdeen, United Kingdom; 3 Department of Biology and Ecology, Faculty of Science, University of Ostrava, Ostrava, Czech Republic; 4 Plant Biology Section, Cornell University, USA

**Keywords:** *Nannochloropsis*, spliced leader *trans*-splicing, simple sequence repeats, LINE, Stramenopiles

## Abstract

Members of eustigmatophyte algae, especially *Nannochloropsis* and *Microchloropsis*, have been tapped for biofuel production owing to their exceptionally high lipid content. Although extensive genomic, transcriptomic, and synthetic biology toolkits have been made available for *Nannochloropsis* and *Microchloropsis*, very little is known about other eustigmatophytes. Here we present three near-chromosomal and gapless genome assemblies of *Monodopsis* strains C73 and C141 (60 Mb) and *Vischeria* strain C74 (106 Mb), which are the sister groups to *Nannochloropsis* and *Microchloropsis* in the order Eustigmatales. These genomes contain unusually high percentages of simple repeats, ranging from 12% to 21% of the total assembly size. Unlike *Nannochloropsis* and *Microchloropsis*, long interspersed nuclear element repeats are abundant in *Monodopsis* and *Vischeria* and might constitute the centromeric regions. We found that both mevalonate and nonmevalonate pathways for terpenoid biosynthesis are present in *Monodopsis* and *Vischeria*, which is different from *Nannochloropsis* and *Microchloropsis* that have only the latter. Our analysis further revealed extensive spliced leader *trans*-splicing in *Monodopsis* and *Vischeria* at 36–61% of genes. Altogether, the high-quality genomes of *Monodopsis* and *Vischeria* not only serve as the much-needed outgroups to advance *Nannochloropsis* and *Microchloropsis* research, but also shed new light on the biology and evolution of eustigmatophyte algae.

**Significance**

Our current knowledge of eustigmatophytes mostly comes from the biofuel algae *Nannochloropsis* and *Microchloropsis*. Here we generated three high-quality genomes of *Monodopsis* and *Vischeria* that are sister to *Nannochloropsis* + *Microchloropsis*. We uncovered an extremely high prevalence of simple repeats in these genomes and found evidence of spliced leader *trans*-splicing. These new genomic resources will greatly facilitate future research to better understand the biology of eustigmatophytes, and to better capitalize on their translational potential.

## Introduction

The diversity of algae is vast but largely unexplored. Despite their often inconspicuous nature, algae have played pivotal roles in Earth’s biogeochemical cycles (de Vargas et al. 2015), and some might hold the key to sustainable bioenergy production (Radakovits et al. 2010; Jagadevan et al. 2018). Eustigmatophytes (Class Eustigmatophyceae), a lineage in Ochrophyta (Stramenopiles), are single-celled coccoid algae that can be found in freshwater, soil, and marine environments (Eliáš et al. 2017). The phylogeny and taxonomy of this group have only been recently clarified (Fawley et al. 2014, 2015; Eliáš et al. 2017; Ševčíková et al. 2019; Amaral et al. 2020). To date, there are around 20 genera and 189 species described according to AlgaeBase (Guiry MD and Guiry GM 2021), although this classification substantially underestimates the actual diversity of the class (Fawley et al. 2021).

**Fig. 1.**
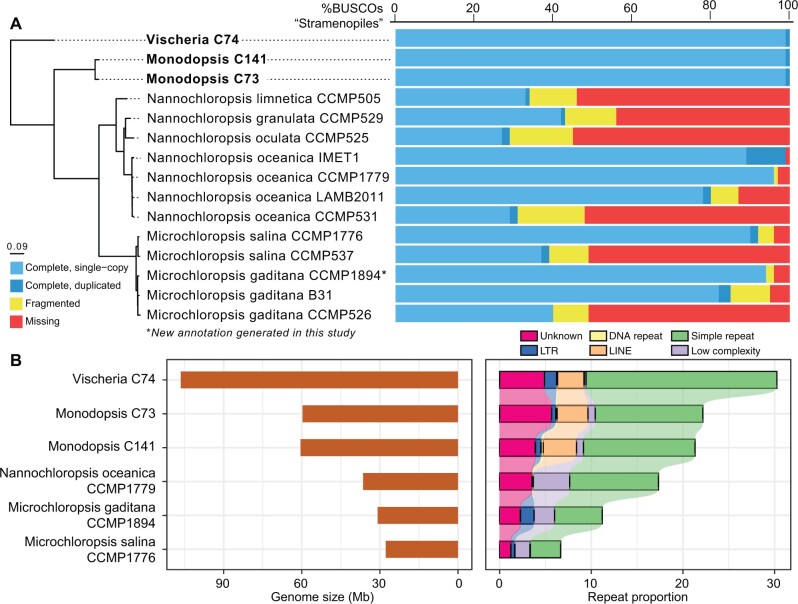
Comparisons of eustigmatophyte genomes. (*A*) The three genomes reported here (in bold) have the highest BUSCO proteome completeness scores compared with the currently available *Nannochloropsis/Microchloropsis* genomes. The “Stramenopile” data set (*n* = 100) was used in the BUSCO analyses. The phylogeny on the left was based on 1,302 single-copy loci, and all branches receive bootstrap support of 100. The rooting was determined by OrthoFinder, which is consistent with the published phylogenies (Ševčíková et al. 2019). (*B*) Overall genome size (left panel) correlates well with repeat content (right panel). Significant expansions of simple repeats and LINEs are evident in *Vischeria* and *Monodopsis* genomes.

The eustigmatophytes that have garnered the most attention are undoubtedly *Nannochloropsis* and the recently segregated *Microchloropsis* (Fawley et al. 2015). Many *Nannochloropsis* and *Microchloropsis* species are capable of producing a staggering amount of lipids, up to 60% of the total dry weight (Eliáš et al. 2017). Because of this, as well as their fast growth rate, much research effort has been devoted to developing *Nannochloropsis* and *Microchloropsis* as an industrial biofuel alga (Eliáš et al. 2017; Jagadevan et al. 2018). The genomes of most *Nannochloropsis* and *Microchloropsis* species, and in some cases multiple strains of species, have been sequenced (Pan et al. 2011; Radakovits et al. 2012; Vieler et al. 2012; Corteggiani Carpinelli et al. 2014; Wang et al. 2014; Schwartz et al. 2018; Brown et al. 2019; Guo et al. 2019; Ohan et al. 2019; Gong et al. 2020). However, only a few assemblies have reached high contig continuity and completeness (fig. 1*A*). In addition, tools for genetic transformation, gene editing, and marker-less trait-stacking have also been developed (Radakovits et al. 2012; Vieler et al. 2012; Wei et al. 2017; Poliner et al. 2018, 2020; Verruto et al. 2018; Naduthodi et al. 2019; Osorio et al. 2019). The applications of these tools and resources have resulted in substantial improvements of lipid production in *Microchloropsis* (previously *Nannochloropsis*) *gaditana* (Ajjawi et al. 2017).

Relatively little is known about the genome structure of eustigmatophytes beyond *Nannochloropsis/Microchloropsis*. To date, most of the research on other eustigmatophytes has focused on the organellar genomes (Ševčíková et al. 2016, 2019; Yurchenko et al. 2016; Huang et al. 2019) and the association with a novel endosymbiont *Candidatus* Phycorickettsia (Yurchenko et al. 2018). Despite many interesting findings that have emerged from these studies, the lack of sequenced genomes throughout eustigmatophytes is limiting further research. Recently, a draft genome of *Eustigmatos* sp. was published as a part of large-scale survey of algal genomic diversity (Nelson et al. 2021). This assembly, however, was fragmented (contig N50 = 102 kb) and was not annotated.

Here we report three near-chromosomal genome assemblies of *Monodopsis* spp. (C73, C141) and *Vischeria* sp*.* (C74). *Monodopsis* is sister to *Nannochloropsis* + *Microchloropsis* in the family Monodopsidaceae (Eustigmatales), and *Vischeria* is a member of the sister family Eustigmataceae, also in the order Eustigmatales (fig. 1*A*; supplementary fig. S1, Supplementary Material online). We carried out comparative studies of repeats and gene space and found evidence of spliced leader *trans*-splicing (SLTS) in these eustigmatophytes. Our results here help to gain a more holistic view on the biology and genomic diversity of eustigmatophytes within the Eustigmatales, expanding beyond what was only known from *Nannochloropsis* and *Microchloropsis*.

## Results and Discussion

### Eustigmatophytes Isolated from Bryophytes

In our ongoing effort to isolate symbiotic cyanobacteria from surface-sterilized bryophyte thalli (Nelson et al. 2019), we have occasionally obtained eustigmatophyte algae instead. DNA barcoding using the 18S rDNA marker indicates all our eustigmatophyte isolates belong to either *Monodopsis* or *Vischeria* (see supplementary fig. S1, Supplementary Material online for the 18S rDNA phylogeny). So far, we have isolates from multiple species of hornworts, liverworts, and mosses, and from diverse geographic locations spread across North America (supplementary table S1, Supplementary Material online). The nature of interaction between eustigmatophytes and bryophytes (if there is any) is unclear. A symbiotic relationship is a possibility, given that similar algal strains have been repeatedly isolated from bryophytes from different locations (supplementary table S1, Supplementary Material online). The recent finding that *Nannochloropsis oceanica* could enter an endosymbiotic relationship with the fungus *Mortierella* (Du et al. 2019) further speaks to the symbiotic competency of eustigmatophytes. On the other hand, both *Monodopsis* and *Vischeria* are common soil algae, and it is possible that they are resistant to our sterilization method and came out as “contaminants.” Future experiments are needed to examine the possible eustigmatophyte–bryophyte interaction.

### Near-Chromosomal Level Assemblies of *Monodopsis* and *Vischeria*

To obtain high quality reference genomes, we generated Illumina short reads and Oxford Nanopore long reads for one *Vischeria* (C74) and two *Monodopsis* strains (C73, C141). The K-mer-based genome size estimates were around 60 and 100 Mb for *Monodopsis* and *Vischeria*, respectively. After filtering, the Nanopore data represented 45–67× coverage with a read length N50 between 13 and 25 kb (supplementary table S2, Supplementary Material online). The assemblies based on Flye (Kolmogorov et al. 2019) are near chromosomal, with the majority of the contigs containing at least one telomeric end (table 1). The telomeric motif is “TTAGGG,” which was also found in *Microchloropsis* (=*Nannochloropsis*) *gaditana* B-31 (Corteggiani Carpinelli et al. 2014). A total of 13,969, 13,933, and 18,346 protein-coding genes were annotated from *Monodopsis* C73, *Monodopsis* C141, and *Vischeria* C74, respectively, all with a 100% Benchmarking Universal Single-Copy Orthologs (BUSCO) (Simão et al. 2015) completeness score against the “Stramenopile” data set. Compared with the published *Nannochloropsis* and *Microchloropsis* genomes, the assemblies we present here are by far the most complete (fig. 1*A*). Interestingly, none of the three genomes contain *Ca.* Phycorickettsia contigs that were previously reported in other eustigmatophytes (Yurchenko et al. 2018).

**Table 1:** Genome Assembly and Annotation Statistics

	*Monodopsis* sp. C73	*Monodopsis* sp. C141	*Vischeria* sp. C74
Assembly size	59.70 Mb	60.47 Mb	106.49 Mb
Contigs, total number	33	43	55
Contigs, with telomere	29	27	40
Contigs, telomere-telomere	22	10	13
Contig N50	2.24 Mb (*n* = 11)	2.04 Mb (*n* = 12)	3.09 Mb (*n* = 14)
Contig N90	1.44 Mb (*n* = 24)	1.12 Mb (*n* = 27)	1.51 Mb (*n* = 33)
Predicted protein-coding genes	13,969	13,933	18,346
BUSCO, genome assembly	96%	99%	98%
BUSCO, predicted genes	100%	100%	100%

Note.—The “Stramenopile” data set (*n* = 100) was used in the BUSCO analyses.

**Fig. 2.**
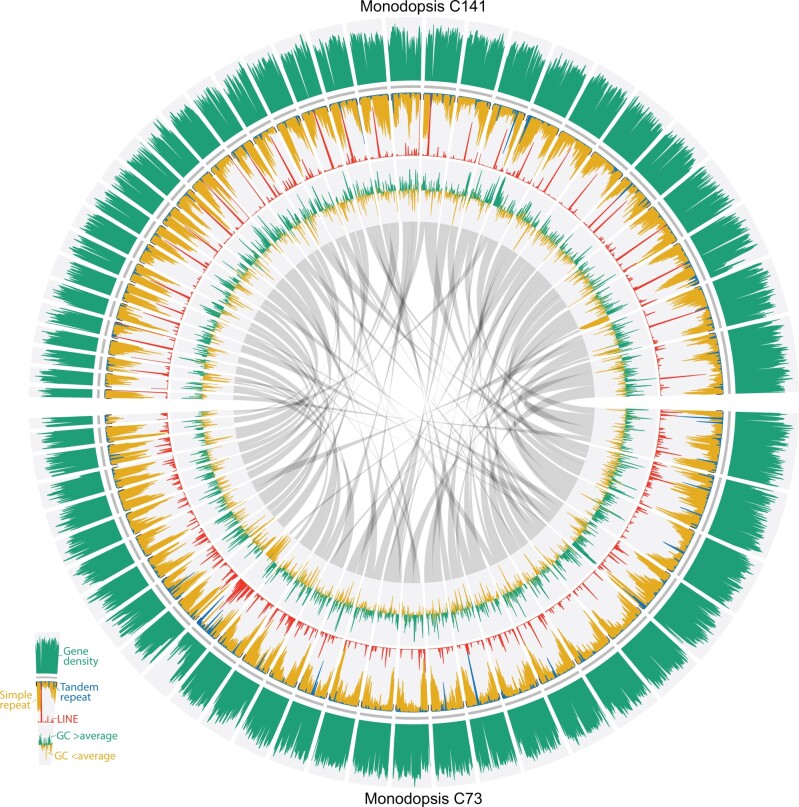
Structures of the two *Monodopsis* genomes. Simple repeats (in yellow) are particularly abundant toward the ends of chromosomes. LINEs (in red), on the other hand, tend to be locally concentrated in the middle of chromosomes (especially in *Monodopsis* C141) and likely represent centromeric regions. Extensive structural variation can be found comparing the two *Monodopsis* genomes, despite their almost identical 18S sequences. Contigs shorter than 500 kb were not plotted.

To gain a better picture of the genetic diversity, we generated Illumina data for two additional strains: *Monodopsis* C143 and *Vischeria* C101. SNP densities between the *Monodopsis* strains (C73, C141, and C143) ranged from 34 to 44/kb, and 10/kb between the *Vischeria* strains (C74 and C101) (supplementary table S3, Supplementary Material online). It is interesting to note that although the *Monodopsis* strains share nearly identical 18S rDNA sequences (>99.78%; supplementary fig. S1, Supplementary Material online), the genomes exhibit substantial structural and nucleotide differences (fig. 2). This finding echoes earlier reports and indicates that, at least in eustigmatophytes, the commonly used 18S rDNA barcode might not properly reflect the underlying genomic diversity and hence underestimate the species richness (Fawley and Fawley 2020).

### A New Annotation of *Microchloropsis gaditana* Genome

Although three *Microchloropsis gaditana* genome assemblies have been published to date, two of them (B-31 and CCMP526) were based on short-read technologies and therefore had low contig N50 length (40.5 kb for B-31 and 15.3 kb for CCMP526) as well as low BUSCO completeness scores (fig. 1*A*) (Radakovits et al. 2012; Corteggiani Carpinelli et al. 2014). Only the *M. gaditana* CCMP1894 genome was assembled using long reads (Schwartz et al. 2018), but unfortunately its annotation has not been published. Here we used publicly available RNA-seq data and protein evidence to annotate the *M. gaditana* CCMP1894 assembly. This new annotation has a much-improved BUSCO score (94% complete) compared with the previous *M. gaditana* annotations (40% and 85%) (fig. 1*A*).

### Unusually High Percentages of Simple Sequence Repeats

**Fig. 3.**
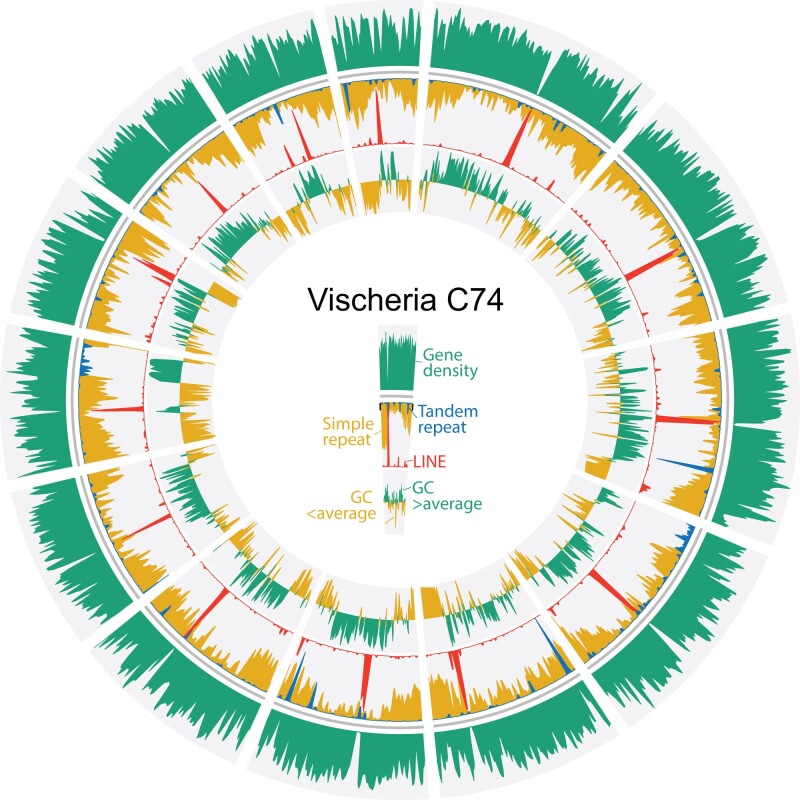
Structure of the *Vischeria* genome. Simple repeats (in yellow) are particularly abundant toward the ends of chromosomes. LINEs (in red), on the other hand, tend to be locally concentrated in the middle of chromosomes and likely represent centromeric regions. For clarity, only telomere-to-telomere contigs were plotted.

*Monodopsis* and *Vischeria* have considerably larger genomes than those of *Nannochloropsis/Microchloropsis*, which can be partly attributed to their higher percentages of repetitive elements (fig. 1*B*). The simple sequence repeats (SSRs) and LINEs are particularly noteworthy. Although LINEs are absent in *Nannochloropsis/Microchloropsis*, they cover around 2.9–3.6% of the *Monodopsis* and *Vischeria* genomes (fig. 1*B*). SSRs have similarly expanded representations, accounting for 11.7–12.2% of the genomic content in *Monodopsis* and 20.8% in *Vischeria* (fig. 1*B*). Although these SSRs can be found throughout the chromosomes, they are particularly enriched toward the chromosome ends (figs. 2 and 3). The frequencies of SSRs observed here are in fact among the highest of all genomes sequenced to date. For example, the human body louse genome (*Pediculus humanus corporis*) had the highest SSR density according to Srivastava *et al.* (2019). When reanalyzed with the same repeat annotation pipeline used here, we found SSRs account for 16.9% of the *P. humanus corporis* genome, making *Vischeria* C74 (at 20.8%) the most SSR-dense genome known to date. Future comparative studies incorporating additional genomes across eustigmatophytes are needed to clarify the impact of such high abundance of SSRs on genome structure and evolution.

### Putative Centromeric Regions That Are Enriched in LINEs

Only a few centromere structures have been experimentally characterized in Stramenopiles. In the oomycete *Phytophthora sojae*, the centromeric regions are particularly rich in the *Copia*-like retroelements (Fang et al. 2020), whereas in the diatom *Phaeodactylum tricornutum*, the centromeres are AT-rich but devoid of repetitive elements (Diner et al. 2017). No putative centromeric region has been identified in *Nannochloropsis/Microchloropsis* to date nor in any other eustigmatophyte. Our analysis of *Monodopsis* and *Vischeria* genomes suggest that their centromeres might be characterized by islands of LINE clusters. The distributions of LINEs in *Monodopsis* and *Vischeria* are highly heterogeneous, usually with a sharp peak toward the middle of a chromosome (figs. 2 and 3). It is likely that such LINE-dense (and gene-poor) regions function as centromeres, but further immunolabeling studies are needed. If confirmed, it would also suggest that *Nannochloropsis/Microchloropsis* might have a substantially different centromere organization given their absence of LINE.

### A Haploid-Dominant Life Cycle

The complete life cycle of eustigmatophytes has not been characterized, and no sexual reproduction has been observed. We found that several meiosis-specific genes are present in *Monodopsis* and *Vischeria*, which is consistent with what was found in *Microchloropsis* (supplementary table S4, Supplementary Material online) (Radakovits et al. 2012; Corteggiani Carpinelli et al. 2014) and suggests eustigmatophytes do have cryptic sexual stages. In addition, we were able to identify homologs encoding flagella-related proteins in both *Monodopsis* assemblies (examples provided in supplementary table S4, Supplementary Material online), despite zoospores never having been documented in *Monodopsis* (but known in *Vischeria*) (Hibberd 1981; Eliáš et al. 2017). Another missing piece of information about the life cycle of eustigmatophytes is the dominant ploidy level. Although earlier genomic studies on *Nannochloropsis* suggested they are monoploid (Pan et al. 2011), no information is available for other members of eustigmatophytes. In order to assess if there is any heterozygosity present in our *Monodopsis* and *Vischeria* strains, we mapped Illumina reads to the respective genomes. We found very few SNPs could be called, and the vast majority of the alternative alleles were supported by low percentages of reads (supplementary fig. S2, Supplementary Material online), suggesting these SNPs were artifacts of residual sequencing and/or assembly errors. Therefore, we infer both *Monodopsis* and *Vischeria* have a haploid-dominant life cycle similar to *Nannochloropsis/Microchloropsis*.

### Terpenoid Biosynthesis Pathways Differ between *Monodopsis*/*Vischeria* and *Nannochloropsis*

Terpenoids are an important class of natural products and have high bioenergy potentials. There are two pathways for terpenoid biosynthesis: the mevalonate pathway (MVA) and the nonmevalonate pathway (MEP). Many Stramenopiles, such as diatoms, have both pathways, whereas all the *Nannochloropsis/Microchloropsis* genomes sequenced to date have only the MEP pathway. Interestingly, in the *Monodopsis* and *Vischeria* genomes, we were able to find intact MVA and MEP pathways present (supplementary fig. S3, Supplementary Material online). The top BLASTP hits of these MVA pathway genes are from other stramenopile species, indicating vertical inheritance of the genes from a stramenopile ancestor instead of horizontal gene transfer into the eustigmatophyte lineage. Because *Nannochloropsis/Microchloropsis* is nested within *Monodopsis* + *Vischeria*, the most likely scenario is that *Nannochloropsis/Microchloropsis* secondarily lost the MVA pathway. This finding highlights the importance of having biodiverse genomes to infer the biology of eustigmatophytes.

### Presence of SLTS and Operons

Our initial analysis of the RNA-seq data revealed a low read mapping rate (∼85%), which is surprising given the high genome completeness and continuity. One possible explanation is the presence of SLTS, which was reported in *M.gaditana* in a patent application (Seshadri et al. 2018). SLTS is a special mRNA maturation process, in which the 5′ end of a pre-mRNA is capped by a spliced leader (SL) sequence that is transcribed from a separate SL locus. The main function of SLTS is to add the necessary 5′ cap to each cistron in a eukaryotic operon (Lasda and Blumenthal 2011). A diverse group of organisms have been shown to have SLTS, including nematodes, cnidarians, and several unrelated protist lineages (Bitar et al. 2013; Krchňáková et al. 2017).

Upon closer inspection with SL detection pipelines, we found evidence of a single SL type in *Monodopsis* and *Vischeria*, and also confirmed the SL previously reported in *M.gaditana* (table 2). The main variants of these SLs were supported by at least 155,671 reads, ensuring confidence in their accuracy (supplementary table S5, Supplementary Material online). All species also possess several minor SL sequence variants at much lower read coverage (supplementary table S5, Supplementary Material online). The main SL variants were *trans*-spliced to 12,313–17,426 AG acceptor sites throughout the genomes. Between 48% and 82% of annotated genes were located within at most 100 bp of an SLTS acceptor site (table 2), and we observed up to 11 SLTS sites per gene (supplementary table S5, Supplementary Material online). This may suggest a complex genome-wide landscape of alternative SLTS in all species, similar to kinetoplastids (Nilsson et al. 2010). The main SL variants were encoded by 24–239 candidate SL RNA genes. Except for *Monodopsis* C141, all species possess at least two dissimilar SL RNA gene variants, which may indicate the presence of pseudogenes (supplementary table S5, Supplementary Material online). Functional SL RNA copies are expected to possess a T-rich region (*Sm* binding motif) that is required for interaction with the splicing machinery (Stover et al. 2006). We found the canonical *Sm* binding motif ATTTTG (Bitar et al. 2013) in six out of 170 SL RNA genes in *Vischeria*, but not in *Monodopsis* and *Microchloropsis* (supplementary table S5, Supplementary Material online). This may indicate that the more recently diverged species *Monodopsis* and *Microchloropsis* have an altered SLTS machinery with different *Sm* motifs, which will require functional molecular studies to elucidate. The secondary structures of the SL RNA genes of all species display at least one major stem loop (supplementary table S5, Supplementary Material online), consistent with SL RNAs in dinoflagellates (Zhang et al. 2007) and tunicates (Ganot et al. 2004), but divergent from the typical three-loop structure in most other organism groups (Krchňáková et al. 2017).

**Table 2:** Summary of SLs Identified in *Monodopsis*, *Vischeria*, and *Microchloropsis*

Genome	SL Sequence (5′–3′)	SL RNA Genes	SLTS Acceptor Sites	% Genes with SLTS Acceptor Sites
*Vischeria* C74	TTTTCAGCCAAGCAACACAAGAAACAAACAACCCACTTCGGGAAACAACAG	170	12,313	48%
*Monodopsis* C73	ATTTTCAGCTAAGACAAAACAAGAACAAAACAACAACCCACTTTCGGGAAACAACAG	25	13,561	62%
*Monodopsis* C141	ATTTTCAGCTAAGACAAAACAAGAACAAAACAAACAACCCACTTTCGGGAAACAACAG	24	12,339	62%
*Microchloropsis gaditana* CCMP1894	AGAATAAACAAACAAAACAATCCCTAAGGGAAAACAACAG	239	17,426	82%

Note.—The main SL sequence variant is presented with the numbers of SL RNA candidate genes, the numbers of SL trans-splice (SLTS) acceptor sites, and the percentage of genes located at most 100 bp downstream of an SLTS acceptor site. Details for SL variants and SL RNA genes are provided in supplementary table S5, Supplementary Material online.

Having established the presence of SLTS in all species, we then tested whether the physical locations of genes that receive SLs may imply the presence of operons. We first reconstructed the 5′ UTRs of gene annotations aided by the identified SLs, which yielded improved annotations for 40–80% of genes (supplementary table S6, Supplementary Material online). Using these improved annotations, we then detected SLs at 36% of genes in *Vischeria*, 58–61% in *Monodopsis*, and 89% in *Microchloropsis*. Requiring downstream genes in operons to receive the SL and intercistronic distances to be no greater than 1,000 bp predicted 682–1,253 operons per species, containing 8–30% of all genes (table 3). Only 21–44 of these operons had intercistronic distances of at most 100 bp (supplementary table S6, Supplementary Material online). Consistent with the much higher SLTS rate, 90% of the putative *Microchloropsis* operons receive the SL at both upstream and downstream genes, whereas *Vischeria* and *Monodopsis* show upstream STLS at only 44–64% of the putative operons. We found no significant (FDR ≤ 0.1) GO or KEGG enrichment in operonic genes compared with the full genomic background, contrary to expectations from other organisms (e.g., Zeller 2010). This may suggest that operon evolution in these species was not necessarily driven by functional coordination of gene expression.

**Table 3:** Summary of Operons Predicted in *Monodopsis*, *Vischeria*, and *Microchloropsis* on the basis of SLTS

Genome	% Genes SL *Trans*-Spliced	Predicted Operons	% Predicted Operons with SLTS Upstream Genes	Predicted Operonic Genes	% Total Genes	Median Intercistronic Distance (bp)
*Vischeria* C74	36%	682	44%	1,408	8%	564
*Monodopsis* C73	61%	1,164	64%	2,442	17%	542
*Monodopsis* C141	58%	1,068	60%	2,216	16%	554
*Microchloropsis gaditana* CCMP1894	89%	1,253	90%	2,765	30%	655

Note.—Predictions required intercistronic distances of at most 1,000 bp and did not require SLTS at upstream operonic genes. The table presents the percentage of genes receiving SL reads, the numbers of operons, the percentage of operons where the upstream operonic gene receives SL reads, the numbers and percentages of operonic genes, and the median intercistronic distances among operonic genes. Details for SL read quantification and operon prediction using alternative criteria are provided in supplementary table S6, Supplementary Material online.

Although these predictions are likely not exhaustive and will require functional validation, they are entirely consistent with other organisms where a single SL is added to both monocistronic and operonic genes, for example, tunicates (Ganot et al. 2004) and platyhelminths (Boroni et al. 2018). Although SLTS has been reported in some algal lineages (Kuo et al. 2013; Roy 2017), our results provide the first insight into the genome-wide landscape of SLTS and putative operons in several eustigmatophyte algae in the order Eustigmatales. Future long-read RNA or cDNA sequencing will help to better define these operons and clarify the functional significance.

## Conclusion

Here we present three high-quality genome assemblies of *Monodopsis* and *Vischeria*. We found that in many aspects, *Monodopsis* and *Vischeria* genomes are substantially different from those of *Nannochloropsis/Microchloropsis*. For instance, *Monodopsis* and *Vischeria* genomes are two to three times larger, and boast one of the highest proportions of simple repeats among sequenced eukaryotic genomes. The centromeric regions in *Monodopsis* and *Vischeria* might be made up by LINE repeats, which are notably absent in *Nannochloropsis/Microchloropsis*. In addition, although *Nannochloropsis/Microchloropsis* lacks the MVA pathway for terpenoid biosynthesis, both MVA and MEP are present in *Monodopsis* and *Vischeria* and likely represent the ancestral state.

We also identified important features that are shared among these eustigmatophyte genomes in the order Eustigmatales. Notably, our finding and the initial characterizations of SLTS unraveled a new aspect of eustigmatophyte biology. We anticipate our new genomic data and associated analyses will greatly facilitate future research to better understand the biology of eustigmatophytes, and to better capitalize on their translational potential.

## Materials and Methods

### Strain Isolation

The three *Monodopsis* (C73, C141, and C143) and two *Vischeria* (C74 and C101) strains sequenced here were isolated from surface-sterilized bryophytes. The localities can be found in supplementary table S1, Supplementary Material online. We followed the methods outlined in Nelson et al. (2019) for cleaning and sterilizing the bryophyte thalli, as well as for establishing unialgal cultures that grew out from the plants. These new algal cultures are available through UTEX Culture Collection of Algae (accession numbers UTEX 3167–3171).

### Genome Sequencing

We sequenced the genomic DNA on both Oxford Nanopore MinION device as well as Illumina NextSeq500 platform. Nanopore libraries were prepared using the Ligation Sequencing kit (SQK-LSK109), and sequenced on MinION R9 flowcells (FLO-MIN106D) for 60 h or until the flowcells died. We carried out basecalling using Guppy v3.0.3 (https://nanoporetech.com/, last accessed July 2021) with the high accuracy flip–flop mode. For *Monodopsis* C73 and C141 strains, reads shorter than 15 kb were discarded prior to assembly, and for *Vischeria* C74, a threshold of 5 kb was used. For Illumina libraries, we followed the general protocol of Nelson et al. (2019) using the SparQ DNA Frag & Library Prep kit and Adapter Barcode Set A. The libraries were pooled with nine other samples and sequenced on one Illumina NexSeq500 mid-output flowcell (150 bp paired-end) at Cornell Institute of Biotechnology. Reads were trimmed and quality-filtered by fastp v0.20.1 (Chen et al. 2018).

### RNA Sequencing

Cells grown on BG11 solution under 12/12 dark/light cycle and 22 °C were harvested by centrifugation and disrupted by an SPEX SamplePrep 1600 MiniG tissue homogenizer. RNA was extracted using Sigma Spectrum Plant Total RNA kit, and strand-specific RNA-seq libraries were made by YourSeq Duet RNAseq Library Kits from Amaryllis Nucleics. The RNA libraries were pooled with 16 other samples and sequenced on one lane of Illumina NovaSeq6000 S-Prime flowcell (150 bp paired-end). Reads were trimmed and quality-filtered by Trimmomatic v0.39 (Bolger et al. 2014).

### Genome Assembly

We first estimated the genome size based on the K-mer frequency of Illumina reads using MaSuRCA v3.3.2 (Zimin et al. 2013, 2017). To assemble the Nanopore reads, we used Flye v2.4.1 (Kolmogorov et al. 2019) with four iterations of built-in polishing, followed by one round of medaka v0.7.1 (https://github.com/nanoporetech/medaka) processing. The nanopore assemblies were further error-corrected by Illumina reads using pilon v1.23 (Walker et al. 2014) with four iterations. To better assemble the telomeric regions, we used teloclip v0.0.3 (https://github.com/Adamtaranto/teloclip) to recover telomeric nanopore reads that can be aligned and appended to the contig ends. Organellar genomes were assembled separately using either GetOrganelle v1.7 (Jin et al. 2020) with Illumina reads, or Flye with a subset of nanopore reads that mapped to organellar genomes of closely related species. The Flye organellar assemblies were polished by pilon until no correction can be made. Finally, the organellar genomes were BLASTn to the nuclear genome assembly to identify and remove any redundant organellar contigs.

### Repeat Annotation

Our initial repeat analysis revealed a large percentage of simple microsatellite repeats, which caused RepeatMasker (Smit et al. 2015) to make many spurious matches to other repeat classes. To address this, we first identified and masked the simple repeats from the genome using RepeatMasker, before building the custom repeat database with RepeatModeler2 (Flynn et al. 2020). RepeatMasker was then used again to annotate and mask all the repeat classes from the genomes. Tandem repeats were identified separately using Tandem Repeats Finder (Benson 1999).

### Gene Model Prediction

Gene predictions were done by BRAKER2 v2.1.5 (Brůna et al. 2021), integrating both protein and transcript evidence with –etpmode and –softmasking flags on. To provide transcript evidence, we mapped RNA-seq reads to the corresponding genome using HiSAT2 v2.1.0 (Kim et al. 2015). To compile the protein evidence, we first used MAKER2 (Holt and Yandell 2011) to train SNAP (Korf 2004) on *Monodopsis* C73 based on reference-guided transcriptome assembly from Trinity v2.1.1 (Grabherr et al. 2011) and *Nannochloropsis/Microchloropsis* protein records from GenBank. The resulting gene models were then annotated with eggNOG v5.0 (Huerta-Cepas et al. 2019), and only genes with annotations were kept as the protein evidence for BRAKER gene prediction. We used the same approach to annotate *M.gaditana* CCMP1894 genome, with transcript evidence from three publicly available RNA-seq data sets (SRA accession numbers: SRR5152511, SRR5152512, and SRR5152516) and protein sequences from *M. gaditana* B31 and *M. salina* CCMP1776. To filter out spurious gene models from BRAKER2, we removed genes that failed to meet all of the following criteria: 1) a TPM expression level at least 0.001, 2) has functional annotation from eggNOG, and 3) was assigned into orthogroups when including all the focal eustigmatophyte genomes in an OrthoFinder v2.3.12 (Emms and Kelly 2019) run. We used BUSCO v4.0.6 (Simão et al. 2015) to assess the completeness of genome assemblies and annotations with the “Stramenopiles” lineage data set. The final gene sets were functionally annotated (including GO and KEGG) by eggNOG v5.0. KEGG pathways were reconstructed using the KEGG Mapper tool (Kanehisa and Sato 2020).

### Visualization of Genome Structures

We used circos (Krzywinski et al. 2009) to visualize the distributions of genes, repeats, and GC content along the genome assemblies. All the sliding windows had a window size of 50 kb and a step size of 25 kb. Gene and repeat densities were calculated using BEDTools 2.28.0 (Quinlan and Hall 2010). GC content deviations were calculated based on whole genome average, which is 0.4615, 0.4620, and 0.5313 for *Monodopsis* C73, *Monodopsis* C141, and *Vischeria* C74, respectively.

### SNP Calling

For each genome, we used bwa v0.7.17 (Li and Durbin 2009) to map Illumina reads to self as well as to the related genomes. We then use bcftools v1.9 (Li 2011) to call SNPs and keep those with quality over 50 and read depth over 20.

### Phylogenetic Relationship of Currently Available Eustigmatophyte Genomes

We compiled a list of the eustigmatophyte genomes that have annotations available (fig. 1), and used Orthofinder v2.3.12 to infer gene orthology. A total of 1,302 single-copy loci were identified, and protein sequence alignments were done by MAFFT (Katoh and Standley 2013). We then carried out phylogenetic reconstruction using IQ-TREE v2.0.3 (Nguyen et al. 2015) on the concatenated alignment matrix with automatic model selection (Kalyaanamoorthy et al. 2017) and 1,000 replicates of ultrafast bootstrapping (Hoang et al. 2018).

### Identification of SLTS

We identified SLs in the C73, C74, and C141 strains as well as *M.gaditana* CCMP1894 (RNA-Seq library SRR10431616 from SRA) using SLIDR 1.1.4 with distance-based clustering (Wenzel et al. 2021). We relaxed the SL length limit (−x 1.25), required GT/AG splice sites and disabled the *Sm* binding motif filter. Identified SL RNA genes were inspected and aligned using MAFFT v7.407. Secondary sequence structures were inferred using RNAfold Web Server (Gruber et al. 2008). Identified SL *trans*-splice acceptor sites were compared against gene annotations using BEDTools 2.28.0 (Quinlan and Hall 2010).

We then tested whether genome-wide SL *trans*-splicing events may indicate the presence of operonic gene organization using SLOPPR 1.1.3 (Wenzel et al. 2021). Because SLOPPR requires accurate gene annotations, particularly at the 5′ end, we first predicted 5′ UTRs guided by identified SLs using UTRme (Radío et al. 2018), relaxing maximum UTR length to 10,000 bp and maximum UTR ORF length to 400 amino acids. Reads containing at least 8 bp of the SL at the 5′ end were then identified and quantified against transcript annotations using SLOPPR. Operon inference was tested with four intercistronic distance cutoffs (infinity, 1,000 bp, 100 bp, and automatic inference) and did not require upstream operonic genes to be SL *trans*-spliced. The functional annotations (GO, KEGG) of candidate operonic genes were tested for overrepresentation against the genome-wide background using hypergeometric tests in ClusterProfiler 3.14.2 (Yu et al. 2012).

## Supplementary Material

Supplementary data are available at *Genome Biology and Evolution* online.

## Supplementary Material

evab233_Supplementary_Data
