# Supplementary material for: Monodopsis and Vischeria Genomes Shed New Light on the Biology of Eustigmatophyte Algae
Source: Genome Biol Evol. 2021 Oct 19;13(11):evab233. doi: 10.1093/gbe/evab233 (PMC8570151; doi:10.1093/gbe/evab233)
Supplement: evab233_Supplementary_Data [file evab233_supplementary_data.zip › Supplemental_files.pdf]

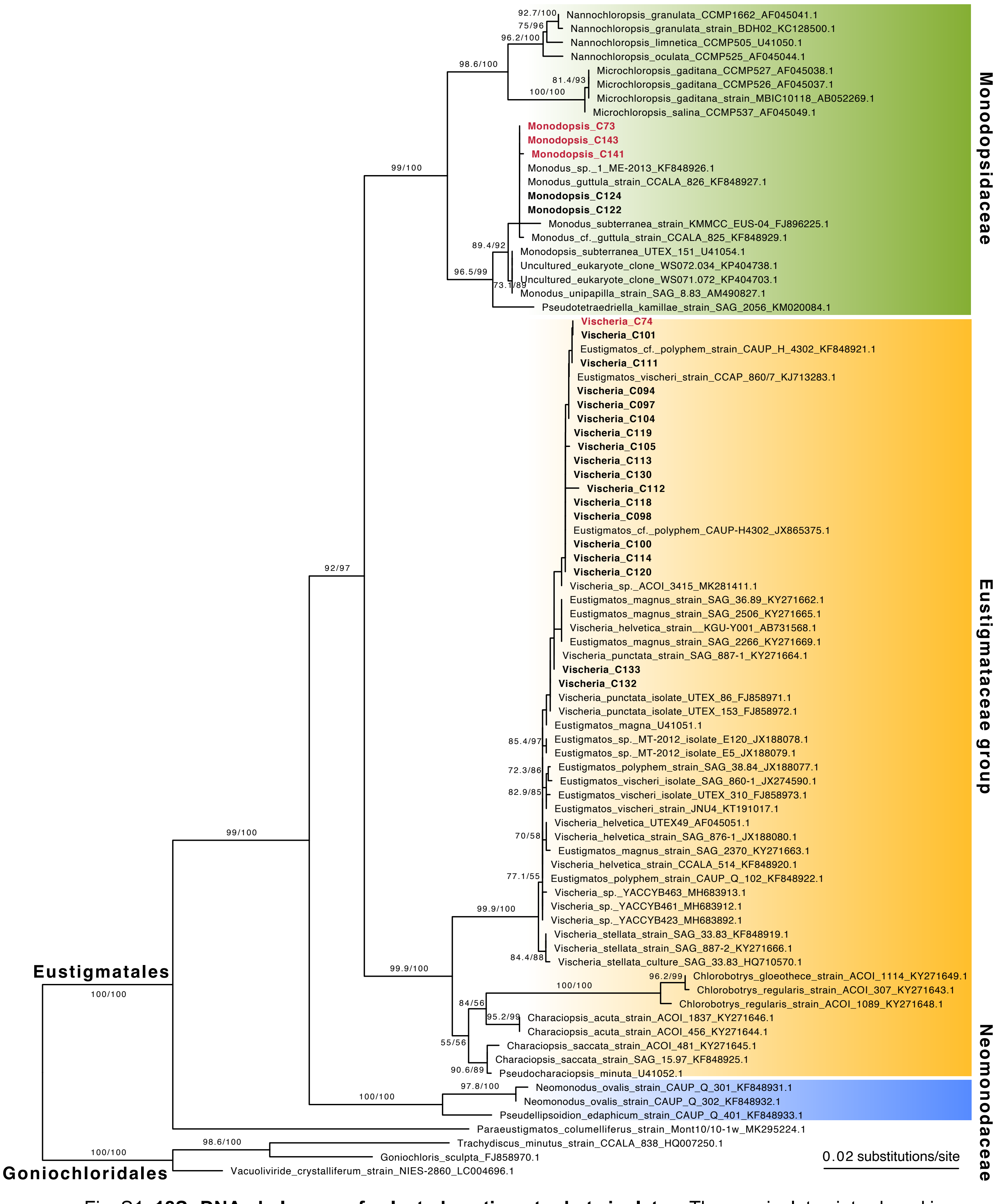

Fig. S1. **18S rDNA phylogeny of selected eustigmatophyte isolates.** The new isolates introduced in this study are in bold, and the sequenced *Monodopsis* and *Vischeria* strains are highlighted in red. The numbers above the branches are: SH-aLRT support / ultrafast bootstrap support, and are omitted if one of the values is below 50.

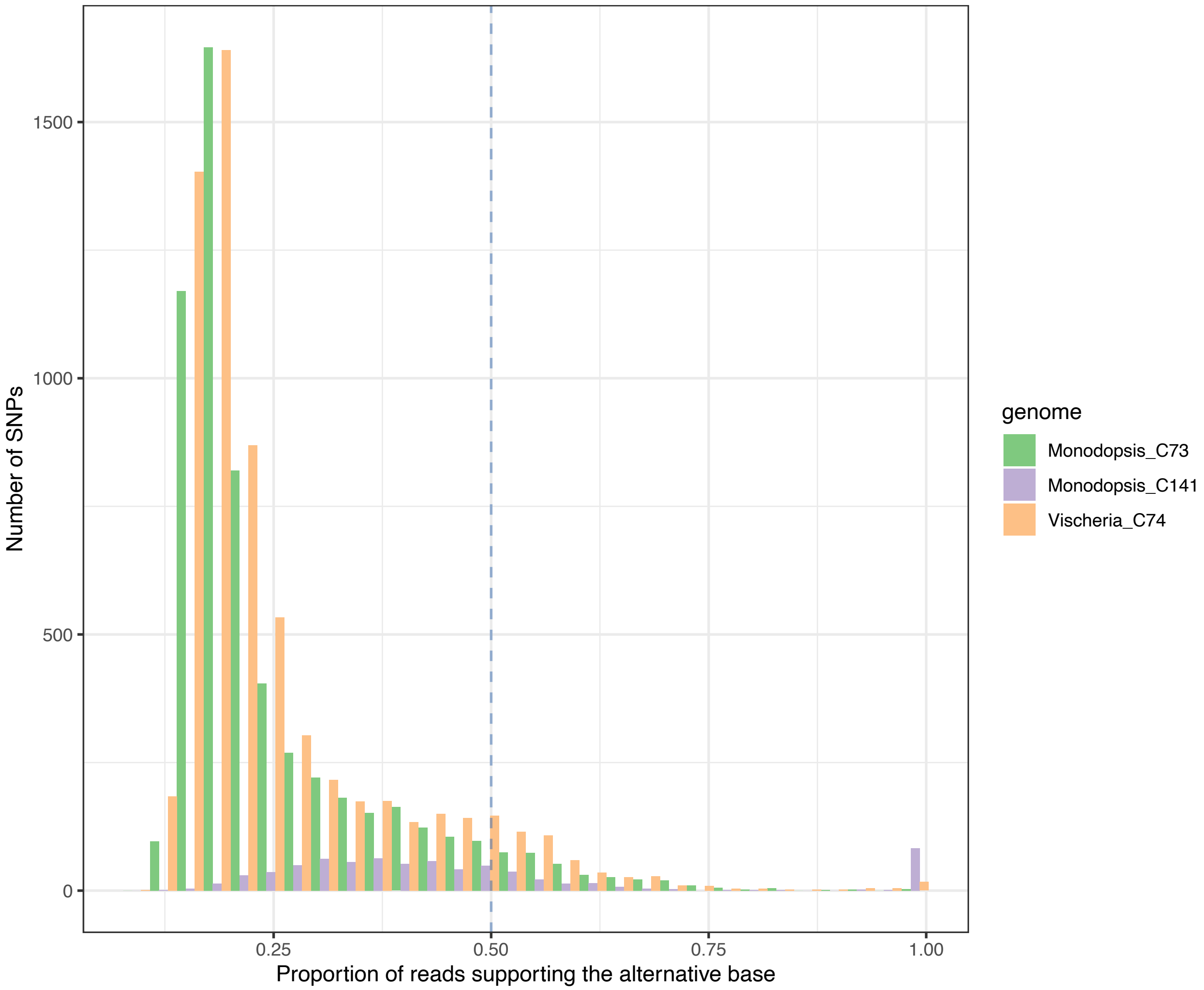

Fig. S2. **The three genomes sequenced here are likely haploid.** Very few SNPs were found while mapping Illumina reads to the respective genomes, and most of the SNPs were supported by a low percentage of reads. In other words, we found no strong evidence of heterozygosity.

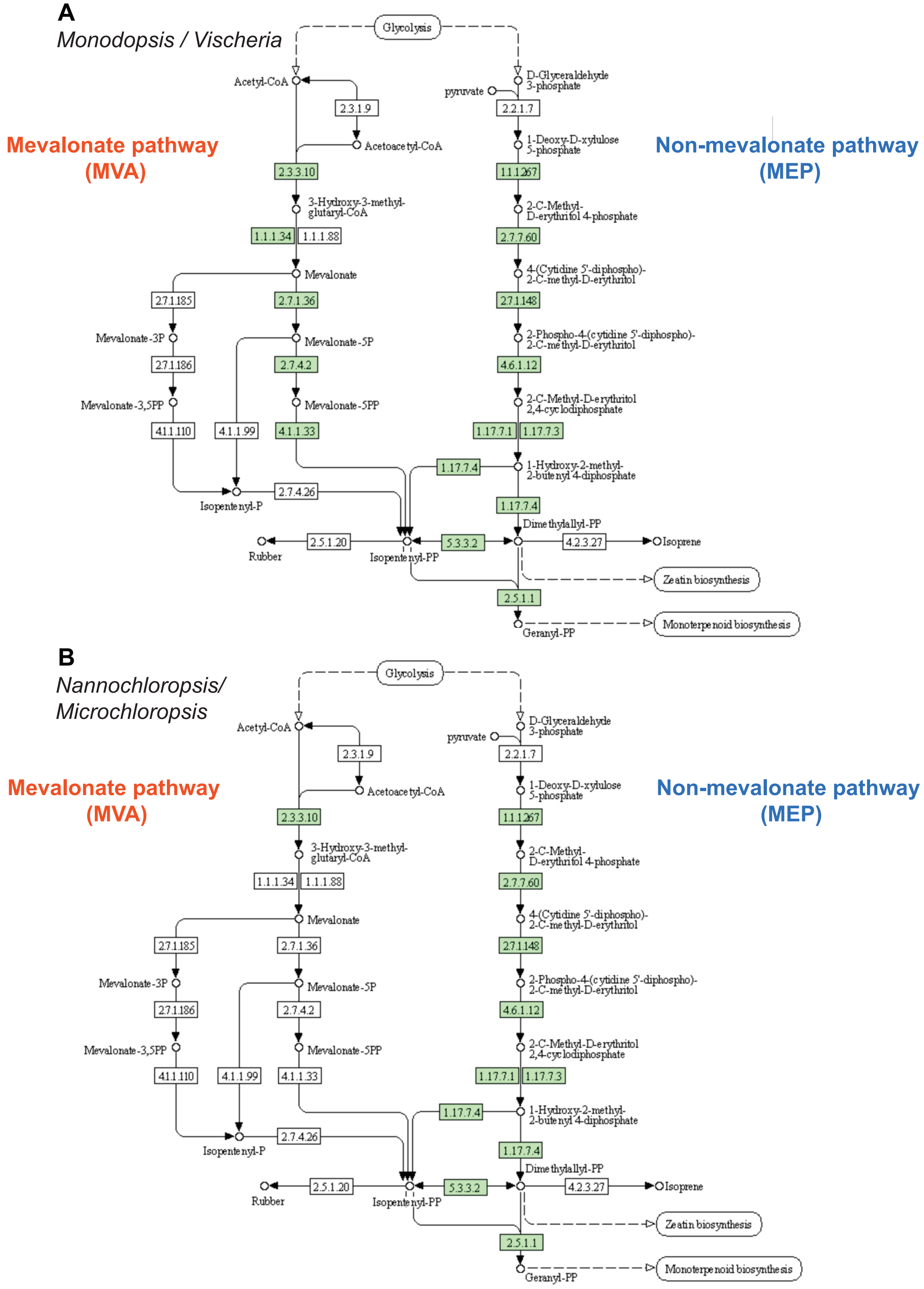

Fig. S3. **Terpenoid biosynthesis pathways.** (A) *Monodopsis* and *Vischeria* genomes have genes encoding for both mevalonate pathway (MVA) and non-mevalonate pathway (MEP). (B) *Nannochloropsis/Microchloropsis*, on the other hand, has only the MEP pathway. The pathway maps were drawn by KEGG mapper.

Table S1. Eustigmatophytes isolated from bryophytes.

Code	Culture Taxon	Isolated from	Host	Collector	Country	State	County	Locality	Habitat	Latitude	Longitude
C-073	Monodopsis sp.	slime cavity	Phaeoceros sp.	Carl Rothfels	USA	California	Mendocino	Van Damme State Park Fern Canyon Trail	sandy bank of small creek	39.27700	-123.74280
C-074	Vischeria sp.	slime cavity	Phaeoceros sp.	Jessica Nelson	USA	North Carolin	Hillsborough	Riverwalk Trail	muddy stream bank	36.07309	-79.10098
C-094 A	Vischeria sp.	slime cavity	Notothylas orbicularis	Fay-Wei Li	USA	New York	Tompkins	Potato Hill State Forest roadside	soil bank	42.34384	-76.26918
C-097 A	Vischeria sp.	slime cavity	Anthoceros agrestis	Fay-Wei Li	USA	New York	Tompkins	Potato Hill State Forest roadside	soil bank	42.34384	-76.26918
C-098	Vischeria sp.	slime cavity	Anthoceros agrestis	Fay-Wei Li	USA	New York	Tompkins	Potato Hill State Forest roadside	soil bank	42.34384	-76.26918
C-100	Vischeria sp.	slime cavity	Notothylas orbicularis	Fay-Wei Li	USA	New York	Tompkins	Potato Hill State Forest roadside	soil bank	42.34384	-76.26918
C-101	Vischeria sp.	slime cavity	Notothylas orbicularis	Fay-Wei Li	USA	New York	Tompkins	Potato Hill State Forest roadside	soil bank	42.34384	-76.26918
C-104	Vischeria sp.	slime cavity	Phaeoceros carolinianus	Fay-Wei Li	USA	New York	Tompkins	Potato Hill State Forest roadside	soil bank	42.34384	-76.26918
C-105	Vischeria sp.	slime cavity	Phaeoceros carolinianus	Fay-Wei Li	USA	New York	Tompkins	Potato Hill State Forest roadside	soil bank	42.34384	-76.26918
C-111	Vischeria sp.	slime cavity	Phaeoceros carolinianus	Fay-Wei Li	USA	New York	Tompkins	Potato Hill State Forest roadside	soil bank	42.34384	-76.26918
C-112	Vischeria sp.	slime cavity	Phaeoceros carolinianus	Fay-Wei Li	USA	New York	Tompkins	Potato Hill State Forest roadside	soil bank	42.34384	-76.26918
C-113	Vischeria sp.	slime cavity	Phaeoceros carolinianus	Fay-Wei Li	USA	New York	Tompkins	Potato Hill State Forest roadside	soil bank	42.34384	-76.26918
C-114 A	Vischeria sp.	slime cavity	Anthoceros agrestis	Fay-Wei Li	USA	New York	Tompkins	Potato Hill State Forest roadside	soil bank	42.34384	-76.26918
C-115	Vischeria sp.	slime cavity	Anthoceros agrestis	Fay-Wei Li	USA	New York	Tompkins	Potato Hill State Forest roadside	soil bank	42.34384	-76.26918
C-118	Vischeria sp.	slime cavity	Notothylas orbicularis	Fay-Wei Li	USA	New York	Tompkins	Potato Hill State Forest roadside	soil bank	42.34384	-76.26918
C-119	Vischeria sp.	slime cavity	Notothylas orbicularis	Fay-Wei Li	USA	New York	Tompkins	Potato Hill State Forest roadside	soil bank	42.34384	-76.26918
C-120	Vischeria sp.	slime cavity	Phaeoceros carolinianus	Fay-Wei Li	USA	New York	Tompkins	Potato Hill State Forest roadside	soil bank	42.34384	-76.26918
C-121	Vischeria sp.	slime cavity	Notothylas orbicularis	Fay-Wei Li	USA	New York	Tompkins	Potato Hill State Forest roadside	soil bank	42.34384	-76.26918
C-122	Monodopsis sp.	slime cavity	Blasia pusilla	Jessica Nelson	USA	New York	Tompkins	Shindagin Hollow State Forest roadside rock face	steep wet rock bank	42.33312	-76.33998
C-124	Monodopsis sp.	slime cavity	Blasia pusilla	Jessica Nelson	USA	New York	Tompkins	Shindagin Hollow State Forest roadside rock face	steep wet rock bank	42.33312	-76.33998
C-130	Vischeria sp.	slime cavity	Phaeoceros carolinianus	Jessica Nelson	USA	New York	Tompkins	Shindagin Hollow State Forest roadside rock face	steep wet rock bank	42.33312	-76.33998
C-132	Vischeria sp.	slime cavity	Phaeoceros carolinianus	Jessica Nelson	USA	New York	Tompkins	Shindagin Hollow State Forest roadside rock face	steep wet rock bank	42.33312	-76.33998
C-133	Vischeria sp.	slime cavity	Phaeoceros carolinianus	Jessica Nelson	USA	New York	Tompkins	Shindagin Hollow State Forest roadside rock face	steep wet rock bank	42.33312	-76.33998
C-141	Monodopsis sp.	slime cavity	Phaeoceros carolinianus	Juan Carlos Villarreal	Canada	Montreal	Saint-Hippoly	Biological Station of the Laurentides	mineral soil	45.98806	-74.00611
C-143	Monodopsis sp.	stellate gemma	Blasia pusilla	Jessica Nelson	USA	New York	Tompkins	Shindagin Hollow State Forest	roadside rock face	42.33312	-76.33998

Table S2. Summary of DNA and RNA sequencing data generated. *Raw data / Size-filtered data.

**Nanopore reads**	**Monodopsis sp C73**	**Monodopsis sp C141**	**Vischeria sp C74**
Total bases	6.502 / 4.013 Gb*	8.335 / 2.747 Gb	5.853 / 4.828 Gb
Read number	938,056 / 162,512	1,569,910 / 121,915	851,498 / 371,407
Average read length	6,931 / 24,695 bp	5,309 / 22,530 bp	6,875 / 12,998 bp
N50 length	18,506 / 25,148 bp	10,215 / 22,208 bp	12,337 / 14,927 bp

**Illumina reads**	**Monodopsis sp C73**	**Monodopsis sp C141**	**Vischeria sp C74**	**Monodopsis sp C143**	**Vischeria sp C101**
Total bases after trimming, genomic	4.224 Gb	4.453 Gb	5.857 Gb	3.520 Gb	6.389 Gb
Total bases after trimming, RNA-seq	7.631 Gb	8.206 Gb	5.309 Gb	-	-

Table S3. Summary of SNP between Monodopsis isolates and Vischeria isolates.

Illumina data source	Reference genome	SNP count	SNP density (no./Kb)
Vischeria_C101	Vischeria_C74	1051266	9.872404645
Monodopsis_C141	Monodopsis_C73	2626993	44.00547586
Monodopsis_C143	Monodopsis_C73	2429648	40.69969597
Monodopsis_C143	Monodopsis_C141	2060523	34.07722284
